# Creating and Evaluating Two Cumulative Developmental Vulnerability Risk Measures.

**DOI:** 10.23889/ijpds.v7i3.1813

**Published:** 2022-08-25

**Authors:** Eric Duku, Molly Pottruff, Magdalena Janus

**Affiliations:** 1 McMaster University

## Objectives

The Early Development Instrument (EDI) is a valid and reliable population-level tool measuring child developmental vulnerability in Kindergarten. The objective of this study was to derive and validate new EDI-based development “cumulative vulnerability” risk indicators using a cumulative risk index approach (Rutter, 1979).

## Approach

The EDI has two main outcome measures: individual domain scores and vulnerability (scoring below a 10% cutpoint). To account for more complexity, we derived two new “cumulative vulnerability” measures. The Mean EDI Domain Score (MEDS) is the mean of the domain scores, and the Total EDI Vulnerability Index (TEVI) is an ordinal summative measure using domain vulnerability indicators. In Study I, we examined the relationship of the MEDS and TEVI measures with neighbourhood-level SES. In Study II, we examined the predictive/explanatory power of the MEDS and TEVI measures with Grade 3 provincial assessments in Ontario, Canada.

## Results

Study I used EDI Kindergarten data from twelve provincial and territorial data collections between 2008 and 2013 in Canada (316,015 children) aggregated to 2,038 customized neighbourhoods. The two new cumulative vulnerability measures worked as expected, with positive association between MEDS and neighbourhood SES (r=0.58), and a negative association between TEVI and neighbourhood SES (r=-0.57). Study II used data from 61,039 Kindergarten children matched between the EDI and Grade 3 EQAO datasets. The predictive/explanatory power of Mean EDI Domain Scores (MEDS; R2=0.11 to 0.15) was twice that of new ordinal summative measure (TEVI; R2=0.06 to 0.08). Interestingly, the predictive power of the TEVI was similar to that of the composite EDI outcome measure, overall vulnerability (vulnerable on one or more domains).

## Conclusion

The MEDS and TEVI work as expected and can be used for research and reporting purposes. More specifically, the TEVI can also be used as a severity metric evaluating the impact of multiple developmental vulnerabilities. It is recommended that further research be conducted to validate the measures with other datasets.

